# Consumption Behaviour towards Branded Functional Beverages among Gen Z in Post-COVID-19 Times: Exploring Antecedents and Mediators

**DOI:** 10.3390/bs13080670

**Published:** 2023-08-10

**Authors:** Teofana Dimitrova, Iliana Ilieva

**Affiliations:** 1 Faculty of Economic and Social Sciences, University of Plovdiv Paisii Hilendarski, 4000 Plovdiv, Bulgaria; 2 Faculty of Economics, University of Food Technologies, 4000 Plovdiv, Bulgaria; i_ilieva@uft-plovdiv.bg

**Keywords:** protection motivation theory, value–belief–norm theory, consumption behaviour, functional beverages, COVID-19

## Abstract

This study investigates the predictors and mediators of branded functional beverage consumption behaviour (CB) within the Gen Z demographic group in Bulgaria. An integrated model based on two widely known psychosocial theories was developed to examine the relationships between the consumers’ personal values within the value–belief–norm theory and the components of the protection motivation theory (PMT). The conceptual framework included two more influencing factors whose impact had not been researched in depth in previous studies concerning CB—namely, the role of media (RM) and branded functional beverage health benefits (HB). An empirical survey based on 435 Gen Z members aged between 16 and 26 years was conducted. Structural equation modelling was used to analyse the proposed hypotheses. The results revealed that the consumers’ personal values were significant predictors of the PMT threat and coping appraisal components, which, in turn, had a significant positive impact on CB. CB was not directly affected by the branded functional beverage health benefits but was indirectly influenced by the HB via purchase intention toward functional beverages and RM. The study highlighted the important role of RM, which directly and indirectly affected CB. The theoretical and practical implications were discussed, and recommendations were given for raising Gen Z’s awareness of the benefits of functional beverages and increasing their consumer acceptance.

## 1. Introduction

The trends over the past years have shown that functional foods (FF) constitute a sunshine segment of the food industry [1]. The primary driving forces for the growth of the functional food market are the emerging interest in the knowledge on the effect of diet on health [2] and the rising medical costs, which provoke people to improve their health via inclusion of functional foods in their diet rather than by spending money on medicine [1]. On the other hand, governments and pharmaceutical companies are also interested in the promising future of functional foods because of the huge healthcare costs associated with patients with chronic diseases, the shorter development times, and the lower development costs in comparison with pharmaceutical products [3]. Chronic non-communicable diseases (CNDs) are the leading global causes of death, being responsible for 74% of the deaths worldwide in 2022 [4]. CNDs are “often caused by a dietary pattern characterised as relatively high in fat, refined sugar, salt, and cholesterol” [5] (p. 1). The Agenda for Sustainable Development adopted in 2015 announced the commitment of all Member States of the United Nations to a one-third reduction in premature deaths from CNDs by 2030 (SDG3). The countries’ progress in this respect was strongly influenced by the coronavirus disease 2019 (COVID-19) pandemic. The COVID-19 direct impact mechanisms on CNDs were found to “include vascular and myocardial injury as well as pancreatic injury, increasing the incidence of new-onset diabetes” [6] (p. 829).

The consumption of foods enriched with functional ingredients (e.g., vitamins, prebiotics and probiotics, polyphenols, plant pigments, phytosterols, minerals, fibre, and fatty acids) could reduce the risk of chronic diseases and improve physical and mental well-being [5]. For 2022, the functional food market was estimated at USD 203.64 billion and was projected to reach USD 229.7 billion by 2023 at an annual growth rate of around 12.8% [7]. Regardless of the good predictions for FF, their development is faced with a number of challenges: lack of an unequivocally recognised definition and regulation dedicated to this food category and a gap between the technological and the nutritional viewpoints [8]. Although several countries have legislation allowing the use and regulation of health claims, the validation process of health claims for FF is still a hard one [9]. The differences among international food regulations increase the difficulties of the food industry in marketing functional food products [3]; the authorisation of any health claims on a food product must be supported by scientific evidence, but communication through complex scientifically formulated terms in the food labelling and advertising could have unintended and opposite effects on consumer acceptance [10].

Japan was the first country to introduce the term “functional foods” to the world in 1984, and it is the only country to have formulated a specific regulatory framework for functional foods. The Japanese government has defined a new product category, Food for Specified Health Uses (FOSHU), as “food containing an ingredient with functions for health and officially approved to claim their physiological effects on the human body” [8] (p. 1). Different attempts have been made to propose a definition of FF by the United States and European countries. The most current version of the definition, according to the Functional Center (FFC), is “natural or processed foods that contain biologically active compounds; which, in defined, effective, non-toxic amounts, provide a clinically proven and documented health benefit utilizing specific biomarkers, to promote optimal health and reduce the risk of chronic/viral diseases and manage their symptoms” [11]. To explore the FF concept from a scientific point of view, the European Commission launched an action on Functional Food Science in Europe (FuFoSE). According to FuFoSE, “food can be regarded as functional if it is satisfactorily demonstrated to affect beneficially one or more target functions in the body, beyond adequate nutritional effects, in a way that is relevant to either improved stage of health and well-being and/or reduction of risk of disease”, which has been the most widely cited definition of functional food in previous studies [5] (p. 2). Despite the fact that there is no standardised definition of FF, a simple one acceptable to most food technologists states that they are foods and food components that provide a health benefit beyond basic nutrition [12,13].

The communication of the health-related effects of food products is important for both manufacturers and consumers [14]. Through the use of a statement denoted as a “health claim” (included in the labelling and approved by official government agencies), manufacturers may provide reliable information and proof of efficacy regarding their health-enhancing FF. The health claims help consumers identify the specific health benefits provided by the consumption of FF and encourage them to make adequate food choices. In addition, they could create sensory and hedonic expectations which would influence the future experiences that consumers would have with the FF [3]. Nevertheless, the communication of these health benefits in a way that consumers will find credible and comprehensible [10] depends entirely on manufacturers.

Beverages are the most attractive and desirable functional food category due to their convenience and ability to respond to the consumer demands for size, shape, more facilitated distribution, and storage [15], sensory characteristics, suitability and affordability [16]. In the literature, a functional beverage is defined as “a non-alcoholic drink that consists of non-traditional ingredients, comprising herbs, minerals, amino acids, vitamins, or additional raw fruit or vegetable ingredients, including probiotics, and is claimed to provide specific health benefits beyond those supplied by any normal food sources” [17] (p. 270). The size of the global functional beverage market is expected to grow from USD 131.47 billion in 2022 to USD 147.7 billion in 2023 at a compound annual growth rate of 12.4%. Asia-Pacific and Western Europe dominated the functional beverage market in 2022 [18]. The functional beverage (FB) segment includes various types of products, among which energy drinks (with caffeine, taurine, glucuronolactone, sugar, B vitamins, and herbal extracts), sports drinks (e.g., isotonic drinks, hypertonic drinks, and hypotonic drinks with different concentrations of salt and sugar), functional bottled water (with vitamins, minerals, antioxidants, amino acids), dairy-based beverages (with prebiotics, probiotics, dietary fibre, phytosterols, monounsaturated fatty acids (MUFA), polyunsaturated fatty acids (PUFA), proteins, minerals, vitamins, etc.), non-dairy-based beverages (prepared from cereals, millet, legumes, fruit, vegetables, and herbs with bioactive compounds, including vitamins, minerals, antioxidants, ω-3 fatty acids, dietary fibre, prebiotics, and probiotics), and others [2,19,20].

The antecedents of the consumer acceptance of FF have been the subject of interest in numerous studies, and a wide range of influential factors have been announced. [5]. However, there is little previous research on the assessment of vital behavioural constructs for predicting functional beverage consumption, specifically branded functional beverage (BFB) consumption behaviour in the post-pandemic setting [21]. The majority of research attention has focused on the nutritional components and health benefits of this type of beverage [1,2,12,17,20]. However, from a business viewpoint, there is a need for further studies of the factors that have a decisive impact on consumers’ intentions for functional beverage purchase and consumption. They would provide manufacturers and traders with more thorough knowledge of their target markets, of the expediency of investing marketing resources in new product categories, of the communication channels used by consumers, etc. This information may prove essential in the making of strategic decisions concerning entry into new markets or, if necessary, emergency actions in future health crisis situations.

Considering this gap identified in the literature, the objective of our study was to explore the antecedents and mediators of post-COVID-19 branded functional beverage consumption behaviour (CB). The focus was on the CB of Gen Z members of working age in Bulgaria (i.e., aged between 16 and 26 years). As pointed out by Gomes et al. [22], each generation shares common characteristics, different from one generation to another. However, compared to other consumer cohorts (e.g., Millennials), Gen Z individuals have grown up with a clear idea of what they want and expect from their future life; that is, these are consumers with an extremely realistic thought pattern and mentality [23]. Furthermore, from a marketing viewpoint, Z-ers constitute a key consumer market for businesses because they are currently young adults, and they will also be potential parents in the future. Hence, the investigation of their behaviour could inform the food companies’ attempts to increase Z-ers’ acceptance of health-based food and “to reduce the negative influence of unhealthy food habits on future generations” [24] (p. 2).

The COVID-19 pandemic has arguably been an enormous global shock to health, economies, and daily life [25]. The countries were faced with major challenges in this extraordinary situation: collapses of their healthcare systems, including workforce shortages; poorly developed primary and preventive care; low health expenditure [26]; colossal disruption in the supply chain networks of medicines, masks, ventilators, testing kits, and commodities required for daily care; high mortality rates among patients with concomitant diseases [27]; job loss; imposition of lockdown and social distancing measures such as closing schools, stores, restaurants, and bars and prohibiting public activities [28]. All companies, regardless of the size and type of their business activities, had to adapt their business models to the new normality, taking into consideration changing consumer behaviour [29].

The pandemic exacerbated some of the well-known, long-standing problems in the healthcare system in Bulgaria: rising morbidity, an insufficient number of hospital beds, a low rate of COVID-19 vaccination, and—above all—a high mortality rate [30]. After initially having low levels of SARS-CoV-2 infections for much of the year, Bulgaria experienced a major epidemic surge at the end of 2020, which caused the highest recorded excess mortality in Europe, among the highest in the word. Subsequently, the country experienced three more major waves, in March–April 2021, in the last few months of 2021, and early in 2022 [31].

Several market research reports show that the COVID-19 crisis reinforced the importance of health and wellness and increased the demand for FB that provide functional benefits, such as immune health, hydration, fluid maintenance, and handling oxidative stress after recovery from COVID-19 [32,33]. Nevertheless, there is a shortage of empirical research that could provide an answer to the question of why consumers prefer functional beverages when they want to enhance their overall health and immunity and what guides them in their decision to purchase or not to purchase such products, especially in the post-pandemic scenario [34].

To successfully complete our objective, the partial least squares structural equation modelling (PLS-SEM), and the SmartPLS 4 software were used to investigate the hypothesised associations. An online/paper-based questionnaire and non-probability-sampling techniques were applied to conduct the survey, combining snowball sampling, convenience sampling and volunteer sampling. A total of 435 valid responses were gathered from participants from all regions of Bulgaria between March 2023 and April 2023.

The rest of the article is structured as follows: Section 2 presents the literature review and the developed research model, which integrates basic constructs from two influential psychosocial theories: the value–belief–norm (VBN) theory and the protection motivation theory (PMT). Section 3 concentrates on the methodology, and Section 4 illustrates the results. Section 5 discusses the findings, taking into account what has been achieved in previous research. Section 6 presents the limitations and conclusions for future research of CB.

## 2. Literature Review and Research Model

Numerous research studies have been dedicated to the determinants of consumer acceptance of functional foods over the past 20 years, including general acceptance, willingness to pay, willingness to buy, willingness to try, FF perceptions, FF consumption, purchase intention, and choice of functional foods [5]. The most frequently discussed psychological and behavioural characteristics of consumers that feature among the factors influencing food choices include awareness, attitudes, motivations, willingness, beliefs, health consciousness, knowledge, lifestyle, etc. [5,35]. For instance, Krystallis et al. [36] reported that health enhancement and health risk prevention through appropriate dietary choices were the most important motives of functional food purchases. Along the same line, FF market reports indicate that the predictors of functional food consumption are related to consumers‘ health motivation, the perceived diet effectiveness of products, and knowledge about nutrition [37]. Similarly, Azzurra and Paola [38] found that consumers purchased functional and organic foods mainly for health reasons. According to the findings of Szakály et al. [39], there was a significant relationship between lifestyle, health behaviour, and the preference for functional food products. In contrast, Barauskaite et al. [13] argued that the consumers’ choice of FF could also be driven by less health-related hedonic or social motives, such as a tendency for indulgence vs. self-control or the motivation to impress and show off. They demonstrated first that the consumers’ tendency towards conspicuous consumption was positively related to both functional food category evaluation in terms of its distinctiveness and self-reported FF purchase rates and second that the descriptive normative susceptibility to peer influence was positively related to the evaluation of the functional food category distinctiveness. Other determinants that may predict the consumer acceptance of functional foods are the sensory and non-sensory FF features, such as brand, price, package, labelling, benefits presented by the product, perceived fit of carrier/ingredient combination [3,5,8,40,41], and the socio-demographic characteristics of consumers, such as age, sex, personality, income, and education level [36,42,43].

There is a limited amount of research on the predictors of branded functional beverage CB. Still, the efforts of some researchers aimed at filling this research gap need to be mentioned (please refer to [21,34,44]). In their pioneering study, Natarajan et al. [44] used the theory of planned behaviour, the health belief model, and the value–attitude–behaviour model as a basis for the empirical investigation of BFB consumption behaviour in the context of the post-pandemic period. They studied the relationships between several variables, i.e., “the role of the media (information about COVID-19)”, the consumers’ “perceived benefits”, their “interest in healthy foods”, the “BFB purchase intention”, “subjective norms”, and “BFB consumption behaviour post-COVID-19”. Their findings indicated that the perceived benefits significantly affected the BFB purchase intention and BFB consumption behaviour. Furthermore, the role of media positively influenced the BFB purchase intention.

This study followed the appeal of Natarajan et al. [21,44] for reworking the suggested research frameworks and conducting behavioural studies that would encompass food consumers in different parts of the globe. On the basis of the theoretical background of the consumption of functional foods and beverages, we decided to study the connections between the basic constructs of two prominent theories, VBN and PMT, in order to investigate the consumption behaviour towards BFB in Bulgaria. Our research model included a total of eight CB antecedents: egoistic value, altruistic value, perceived vulnerability, perceived severity, perceived self-efficacy, BFB purchase intention, role of media (information about COVID-19), and BFB health benefits.

### 2.1. Consumers’ Personal Values According to Value–Belief–Norm Theory

The VBN theory [45] integrates Schwartz’s consumers’ personal values (PVs) theory, the norm activation model (NAM) theory [46,47], and the New Environmental (or Ecological) Paradigm [45]. The VBN model represents a decision-making process that hierarchically describes how values, beliefs, and personal norms influence individual behaviour. In it, PVs play a central role [48]. Building on the concepts of Schwartz’ self-transcendence values and self-enhancement values, Stern [45] conceptualised PVs via three types of value orientations: altruistic, biospheric, and egoistic. Altruistic values are concerned with the welfare of society (i.e., people with altruistic values act on behalf of others without expecting any kind of personal benefits). Egoistic values, on the other hand, allude to the fact that an individual acts for himself/herself to avoid harm (i.e., acts for self-benefits) [49]. Biospheric values are concerned with environmental protection (i.e., prioritising nature) [48]. Although VBN has been primarily applied to environmental research, it has also been recently used within more socio-cultural contexts [50]. Several studies have made use of this model to predict organic food consumer behaviour [51,52]. In view of the fact that the new functional food ingredients are from natural sources [16] and that similarly to organic foods [53], the healthiness of FF could also determine consumers’ preferences, we have reasons to believe that VBN can be used as a conceptual framework to understand BFB consumption behaviour.

Following Yadav’s [52] conceptualisation, this study considers values as being altruistic and egoistic, which can be regarded as an environmental concern and a health concern, respectively. Previous research (focused on consumer adoption of eco-friendly behaviour, in particular) also used the approach of distinguishing between these two consumers’ personal values [49,54,55,56]. In this sense, environmental concern “can be understood as altruistic in nature, as the individual performs these behaviours of protecting the natural environment”. Also, the health concern of an individual can be seen as “the pro-self (concern for self or to their family) concept, so it can be understood as egoistic in nature” [52] (p. 93). Our review of the literature has shown that no study has considered egoistic and altruistic values together in order to explain BFB consumption behaviour, especially in post-COVID-19 times.

The current literature has indicated that the health concerns of customers can influence their purchasing decisions towards green products or organic food [54]. Therefore, we argue that seeking self-benefits (egoistic values), such as better health, are likely to motivate consumers to adopt BFB. The health concern could provoke individual intention to self-protect from danger (for instance, COVID-19 or a perceived CND threat). Thus, Sun et al. [55], for instance, found that awe induced by COVID-19 positively affected green consumption behaviour. In the post-COVID-19 context, consumers with high health concern levels will probably share the view that COVID-19 still poses a serious risk to their health. Contrarily, consumers with altruistic values (via the prosocial self-schema and environmental consciousness line they follow) will maintain that COVID-19 is still a threat to all individuals, not to them personally. In this sense, we accept that both altruistic and egoistic values will positively influence PMT threat and coping appraisal variables, including vulnerability, perceived severity, and perceived self-efficacy to COVID-19. Moreover, consumers with high levels of altruistic and egoistic values would react positively to the information about COVID-19 (role of media). And finally, on the basis of the findings of Lavuri [56], which indicated that both altruistic and egoistic values had a significant positive impact on green buying intentions, we argue that PVs will exert a direct effect on BFB purchase intention. Hence, we offer the following hypotheses:

**Hypothesis 1** **(H1).** *Egoistic values positively influence their (a) perceived vulnerability, (b) perceived severity, (c) perceived self-efficacy, (d) BFB purchase intention, as well as (e) the role of media.*

**Hypothesis 2** **(H2).** *Altruistic values positively influence their (a) perceived vulnerability, (b) perceived severity, (c) perceived self-efficacy, (d) BFB purchase intention, as well as (e) the role of media.*

### 2.2. Protection Motivation Theory Components

The PMT, proposed as a fear-appeal theory by Rogers [57], and its revised version [58] have been successfully applied to many health promotion activities and healthy lifestyle enhancing behaviours [59]. Recently, the PMT has also been used in the COVID-19 context to explore the factors affecting consumers’ panic buying [60], the influence of tourists’ COVID-19-related threat and coping appraisal factors of PMT on the information-seeking attitude [61], and the restaurant patron’s intention to practice self-protection [62]. According to the PMT, people use both threat appraisal and coping appraisal in order to protect themselves from harm [63]. Since the PMT has three cognitive processes (sources of information, cognitive mediating process, and coping modes) with many constructs, most of the previous research used only part of the constructs [59]. In this study, we chose three threat and coping appraisal components of the PMT to predict the BFB consumption behaviour. To us, consumers’ “perceived vulnerability” or susceptibility can be defined as “an individual’s assessment of their personal vulnerability and exposure to risk” [61] (p. 3). “Perceived severity” is defined as “the seriousness that an individual associates with the consequences in the event of a health crisis” [60] (p. 2). “Perceived self-efficacy” refers to “a person’s assessment of the degree to which he/she is capable of carrying out a recommended coping action” [61] (p. 3). We regard the protection motivation, or “BFB purchase intention”, as “a situation wherein consumers tend to buy a BFB product in a particular situation, in this context, the post-COVID-19 settings” [44]. The BFB consumption behaviour refers a manifest, observable response in a given situation (in this case, the post-COVID-19 period) to a given target (i.e., BFB) [59].

Cox et al. [63] were the first ones to apply the PMT to perceptions of health-enhancing foods or supplements. Their results demonstrated that perceived self-efficacy was one of the most important predictors of intentions to consume FF. Park et al. [59] also tested the PMT to explore adult consumers’ functional food consumption behaviour. They reported that self-efficacy was the only construct that significantly predicted intention as well as behaviour regarding FF. Nystrand et al. [64] also demonstrated the strong influence of self-efficacy on the intention to buy or consume functional food products. Ryu et al. [62] found that “self-protective intention” was influenced more by the perceived threat of COVID-19, but the actual behaviour of “restaurant visit frequency” and “protective behaviour at restaurants” was influenced more by the positive PMT factors: perceived efficacy or self-confidence about protecting oneself and the expected rewards of dining out. To the best of our knowledge, no prior research is available on the effect of the PMT threat and coping appraisal variables on the role of media (information about COVID-19). A large number of studies have demonstrated that behavioural intention is a dependent variable that plays a vital role in explaining the prediction of consumer behaviour [65,66]. Regarding BFB purchase intention, Natarajan et al. [21,44] reported that the effect of BFB purchase intention on the post-COVID-19 BFB consumption behaviour was the strongest amongst all significant relationships in their structural model. Therefore, we hypothesised that:

**Hypothesis 3** **(H3).** *Consumers’ (a) perceived vulnerability, (b) perceived severity, and (c) perceived self-efficacy positively influence their BFB purchase intention.*

**Hypothesis 4** **(H4).** *Consumers’ (a) perceived vulnerability, (b) perceived severity, and (c) perceived self-efficacy positively influence the role of media.*

**Hypothesis 5** **(H5).** *Consumers’ (a) perceived vulnerability, (b) perceived severity, (c) perceived self-efficacy positively influence post-COVID-19 BFB consumption behaviour.*

**Hypothesis 6** **(H6).** *BFB purchase intention positively influences post-COVID-19 BFB consumption behaviour.*

### 2.3. Role of Media and Health Benefits

Prior research established that “health information, such as the type of added functional ingredients and how they benefit human health, may lead to higher purchase intentions” [5] (p. 8). Consumers may be more willing to accept FF if the perceived health benefits exceed their threat-coping confidence (the perceived health hazard). Therefore, it is of primary importance to FF manufacturers to take actions towards reducing consumer suspicion and mistrust by educating consumers on the health benefits of FF or functional ingredients through ethical and informative marketing [40]. In addition to the provision of health information on product labels, further communication effort is needed via various health-related media sources whether in printed, audiovisual, or electronic form [34]. According to Moors [67], the information on health-enhancing functional foods needs to be easily accessible and reliable as well as supplied by general practitioners, dieticians, and TV programmes about product quality and comparisons. The specific health benefits of FF consumption that may be emphasised are: (1) preventing the replication of SARS-CoV-2 and blocking viruses, (2) alleviation of post-COVID-19 complications (such as skeletomuscular symptoms, gastrointestinal symptoms, and neuronal symptoms) in recovering individuals [68], (3) reducing CND occurrence risk (e.g., cardiovascular diseases and certain types of cancer) [1], and treatment of psychiatric diseases [69]. Savelli and Murmura [24] demonstrated that the perceived benefits of healthy food are direct antecedents of older Z-ers’ behavioural intention. Labrecque et al. [70] provided empirical evidence that consumers’ FF purchase intention was positively correlated with health benefits. Natarajan et al. [44] confirmed the direct and positive relationship of perceived benefits and the BFB purchase intention.

Our assumption was that COVID-19 information on nutritional support (“role of media”) would have a positive effect on both the BFB purchase intention and the BFB consumption behaviour, although previous research failed to confirm the existence of such significant influence of the role of media on these two constructs [44]. Additionally, we assumed that the BFB purchase intention, the role of media (information about COVID-19), and post-COVID-19 BFB consumption behaviour were effectively influenced by BFB health benefits. Hence, the following hypotheses were proposed:

**Hypothesis 7** **(H7).** *The role of media positively influences (a) BFB purchase intention and (b) post-COVID-19 BFB consumption behaviour.*

**Hypothesis 8** **(H8).** *BFB health benefits positively influence (a) BFB purchase intention, (b) the role of media, and (c) post-COVID-19 BFB consumption behaviour*.

### 2.4. The Mediating Factors: PMT Components and Role of Media

Similarly to prior research on FF consumption behaviour [71], we decided to investigate not only the direct effects of the constructs in our research model but also the indirect effects produced by mediating constructs. Accordingly, we expected that the PMT threat and coping appraisal variables could mediate both the hypothesised relationships of background factors (egoistic and altruistic values) with the role of media, and the purchase intention, respectively, which has not been examined in the functional food context. Furthermore, considering the findings of previous PMT studies [59,62], we assumed that the BFB purchase intention was a mediator between the PMT threat and coping appraisal variables and actual protective behaviours (i.e., post-COVID-19 BFB consumption behaviour). In this line, it is plausible to believe that there are mediation effects of the BFB purchase intention in both the associations of post-COVID-19 BFB consumption behaviour with the role of media, and the BFB health benefits, respectively. Hence, we put forward the following hypotheses:

**Hypothesis 9** **(H9).** *Consumers’ (a) perceived vulnerability, (b) perceived severity, and (c) perceived self-efficacy mediate the relationship between egoistic values and the role of media.*

**Hypothesis 10** **(H10).** *Consumers’ (a) perceived vulnerability, (b) perceived severity, and (c) perceived self-efficacy mediate the relationship between altruistic values and the role of media.*

**Hypothesis 11** **(H11).** *Consumers’ (a) perceived vulnerability, (b) perceived severity, and (c) perceived self-efficacy mediate the relationship between egoistic values and the BFB purchase intention.*

**Hypothesis 12** **(H12).** *Consumers’ (a) perceived vulnerability, (b) perceived severity, and (c) perceived self-efficacy mediate the relationship between altruistic values and the BFB purchase intention.*

**Hypothesis 13** **(H13).** *The BFB purchase intention mediates the relationships between (a) perceived vulnerability, (b) perceived severity, and (c) perceived self-efficacy and post-COVID-19 BFB consumption behaviour.*

**Hypothesis 14** **(H14).** *The BFB purchase intention mediates the relationship between the role of media and post-COVID-19 BFB consumption behaviour.*

**Hypothesis 15** **(H15).** *The BFB purchase intention mediates the relationship between BFB health benefits and post-COVID-19 BFB consumption behaviour.*

Furthermore, there is a possibility of indirect effects of the role of media on the relationships of PMT threat and coping appraisal factors with BFB purchase intention and post-COVID-19 BFB consumption behaviour, respectively. It may also be observed that the information about COVID-19 acts as a mediator in both the associations of BFB health benefits with protection motivation (BFB purchase intention) and actual protective behaviours. Therefore, we offer the following hypotheses:

**Hypothesis 16** **(H16).** *The role of media mediates the relationships between (a) perceived vulnerability, (b) perceived severity, (c) perceived self-efficacy and BFB purchase intention.*

**Hypothesis 17** **(H17).** *The role of media mediates the relationships between (a) perceived vulnerability, (b) perceived severity, (c) perceived self-efficacy and post-COVID-19 BFB consumption behaviour.*

**Hypothesis 18** **(H18).** *The role of media mediates the relationship between BFB health benefits and BFB purchase intention.*

**Hypothesis 19** **(H19).** *The role of media mediates the relationship between BFB health benefits and post-COVID-19 BFB consumption behaviour.*

**Figure 1:**
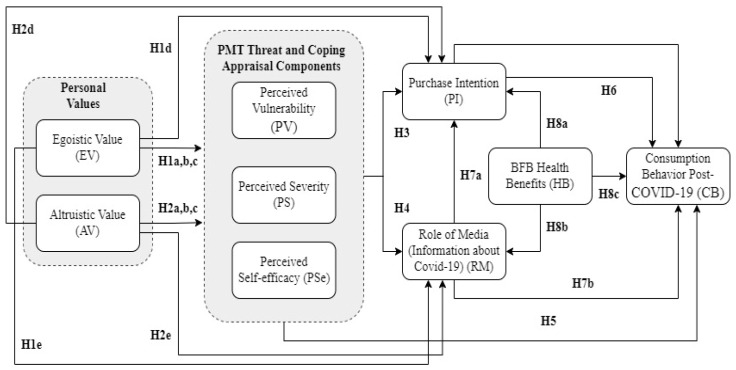
Research model.

The hypotheses and research model are depicted in Figure 1:

## 3. Methodology

### 3.1. Measurement Instrument

The questionnaire of the survey was composed of two sections. The first section aimed to gather information about the socio-demographic factors of the participants (gender, age, level of education, personal income, and place of residence), while the second part was designed to obtain data related to the variables considered. The survey questionnaire items were adopted and adapted from previously tested scales, one of which (EV2) was reverse-scored (please refer to Abbreviation part for the abbreviations of the items). All scales were measured on a five-point Likert scale (as done in the sources cited in Table 1) except for the BFB health benefits, which were measured on a five-point semantic differential scale.

The pilot test with 44 respondents was conducted to ensure the survey questions were easy to understand and appropriate for the participants’ level of comprehension. Furthermore, the reliability and validity of the constructs were assessed through Cronbach’s alpha (α > 0.70), construct reliability (CR > 0.70) and analysis of variance extracted (AVE > 0.50). All results were satisfactory, and no changes to the questionnaire were needed.

### 3.2. Sample and Data Collection

For the main survey, the questionnaire was complemented with photos of well-known FB brands in Bulgaria in five product categories: energy drinks, functional bottled water, dairy-based beverages, and non-dairy-based beverages. Thus, unlike previous studies focused on one or a smaller number of FF, this research opens the spectrum to a greater number of products in order to provide a holistic view of the consumption of this food product type. With a view to the precise definition of the Generation Z age group, we adopted the classification put forth by Agrawal et al. [72], who claimed that anyone born between 1997 and 2012 was referred to as Gen Z. In this line, all individuals aged between 11 and 26 years can be considered Gen Z members. However, we defined Gen Z as those between 16 and 26 years old as the target audience of this study since the working age in Bulgaria begins at 16. The group comprises young people who can start a career and have disposable income as working people able to make their own decisions regarding the purchase of certain products. According to the data of the National Statistical Institute in the Republic of Bulgaria (NSI), individuals aged between 16 and 26 constituted 65.27% of Gen Z in Bulgaria as of 31 December 2022.

The data were collected through an online and a paper-based questionnaire between 15 March and 15 April 2023. Visits to supermarkets and shopping malls were organised to cover the respondents’ opinion through the paper questionnaire, whereas the online version of the questionnaire was made available to Generation Z in Bulgaria by email, social networks (Facebook, Instagram, and LinkedIn), and a Google form. Participation was voluntary and anonymous, and the participants were informed about the purpose of the study and gave their informed consent. The completed questionnaires were subjected to a screening process to ensure the data quality.

The collected sample contained responses from 804 participants, 677 of whom habitually bought BFB. Out of the participants who habitually bought functional foods, 471 were young people of Generation Z, the target audience of this study. After the screening process, 435 valid questionnaires were used for analysis. Men accounted for 51.5% and women for 48.5% of the entire population. This sample distribution by gender corresponded to the gender structure of the population included in the 16-to-26-year-old age group in Bulgaria as of 31 December 2022 (according to data provided by NSI). The majority of the respondents (87.8%) had completed secondary education, followed by higher education graduates (9.4%) and people with primary education (2.8%). Regarding personal income, 47.4% of the respondents declared income lower than 780 BGN, i.e., below the minimum monthly wage in Bulgaria as of 1 January 2023. Nearly half of the participants in the survey (48.6%) had their current address in a city with above 100 thousand inhabitants.

The socio-demographic distribution of the survey can be found in Table 2.

The research hypotheses were tested by using structural equation modelling (SEM) and SmartPLS software [73]. SmartPLS is effective software that can estimate complex models with latent variables and provide comprehensive reports. SEM is composed of a measurement model and a structural model. The measurement model assesses the validity of the indicators (items) for each construct, while the structural model tests all the hypothetical relationships between the constructs. The measurement model in the present study is reflective in nature, i.e., the indicators are affected by the latent variable and are interchangeable because they all represent aspects of the same conceptual domain [74].

All procedures and cut-off values were in conformity with the recommendations in the specialised literature [75]. The normality of the data was assessed via skewness and kurtosis tests. On the basis of the results obtained, a conclusion could be made that the respondents’ pattern was considered a normal distribution since both the absolute skewness value and the absolute kurtosis value were less than 1.

Further, the data were checked for common method bias (CMB) by applying Harman’s single-factor test. The single factor only explained 29.3% of the total variance, which was lower than the critical value of 50%, suggesting no CMB issue [76].

## 4. Results

### 4.1. Measurement Model

The estimation of the reflective measurement model was performed through the assessment of the internal consistency reliability and construct validity. The internal consistency reliability was measured using Cronbach’s alpha and composite reliability (CR). Both statistics for the considered constructs were over the required threshold of 0.70. Hence, the construct reliability was established. The construct validity was assessed through convergent and discriminant validity. The convergent validity was estimated using the average variance extracted (AVE), whereas the Fornell–Larcker criterion and heterotrait–monotrait ratio (HTMT) were used to evaluate the discriminant validity. The AVE values of the constructs were greater than the recommended value of 0.5 and the square of the correlation coefficient of the constructs with other constructs. All HTMT values were far below the 0.85 benchmark, once again indicating no lack of discriminant validity. Therefore, the construct validity for the model was established. The reliability and validity analyses have been presented in Table 3 and Table 4.

Finally, the goodness of fit (GoF) for the hypothesised model was 0.44, indicating that the model satisfied the global criterion of 0.3 [77].

On the basis of the results obtained so far, a conclusion can be drawn that the measurement scales adopted for the present study had sufficient psychometric quality and could be used in the next phase of the analysis.

### 4.2. Structural Model

In the context of the structural model evaluation, an important step is to assess the multicollinearity through variance inflation factors (VIF). In the present study, all the VIF values were found to be lower than the threshold of 5, indicating lack of collinearity issues. The significance of the path coefficients was assessed via the bootstrapping method with 5000 resamples and a one-tailed test.

Table 5 summarises the findings of the testing of the hypothesised direct effects. The results demonstrate that 21 out of the 25 proposed direct relationships were supported in the hypothesised conceptual framework, whereas 4 were not.

Alongside the direct relationships, a mediation analysis was also performed (Table 6).

## 5. Discussion and Implications

Our study investigated the antecedents and mediators of post-COVID-19 branded functional beverage consumption behaviour of Gen–Z in Bulgaria. We formulated nineteen (including eleven mediating) hypotheses by testing 25 proposed direct relationships and 25 indirect relationships between the factors. Our findings supported most of the research hypotheses put forward in this study and provided in-depth insights into the analysis of the factors influencing the BFB consumption behaviour of Gen Z, offering novel and additional knowledge to the prior literature.

### 5.1. Direct Effects

All relationships in the first hypothesis (H1a–e) were supported, indicating that the egoistic values (health concern) of Z-ers positively influenced (1) the components of the PMT threat and coping appraisals (consumers’ egoistic value orientations—perceived vulnerability, *β* = 0.166, *p* = 0.000; consumers’ egoistic value orientations—perceived severity, *β* = 0.243, *p* = 0.000; consumers’ egoistic value orientations—perceived self-efficacy, *β* = 0.223, *p* = 0.000), (2) their BFB purchase intention (consumers’ egoistic value orientations—BFB purchase intention, *β* = 0.132, *p* = 0.002), and (3) the reaction to the information about COVID-19 (consumers’ egoistic value orientations—role of media, *β* = 0.091, *p* = 0.020). These findings seemed to be in line with other food-related research [52]. Furthermore, it could be concluded that EV had the greatest effect on PS, followed by PSe, PV, PI, and RM (see Table 4). This result was in conformity with our assumption that the Z-ers looking for self-benefits (egoistic values) would consider COVID-19 to be a threat that is hard to overcome and of greater weight.

The statements in the second hypothesis (H2a–e) were confirmed, except for H1d. The results revealed that the altruistic values (environmental concern) of Z-ers positively influenced the components of the PMT threat and coping appraisals (consumers’ altruistic value orientations—perceived vulnerability, β = 0.310, *p* = 0.000; consumers’ altruistic value orientations—perceived severity, β = 0.299, *p* = 0.000; consumers’ altruistic value orientations—perceived self-efficacy, β = 0.384, *p* = 0.000). Also, the expected positive relation between the Z-ers’ altruistic values and the information about COVID-19 was supported (consumers’ altruistic value orientations—role of media, β = 0.102, *p* = 0.028). The proposed direct relationship between the altruistic values of Z-ers and their BFB purchase intention was not supported (consumers’ altruistic value orientations—BFB purchase intention, *β* = −0.001, *p* = 0.294). This result was surprising, as it statistically contradicted our assumption. It could be attributed to the fact that the BFB purchase intention of Gen Z with altruistic attitudes was not guided by personal benefit, unlike that of consumers with egoistic value orientations. In this sense, it could be a challenge to the FF industry to attract Gen Z, who would react according to their altruistic motives when making their FF choice. The efforts could be concentrated upon the sustainable, environment-friendly manufacture of alternative BFB involving food waste utilisation, e.g., fruit by-products containing vitamins, minerals, essential fatty acids, carotenoids, antioxidants, phytosterol, and fibre [78]. A recent study by Gupta et al. [69] foregrounded the supply of customised value-added products that meet the special needs of consumers as one of the new opportunities for the functional beverage sector.

On the other hand, Smith and Paladino [79] did not confirm the existence of a positive effect of environmental concerns on consumers’ intention to purchase organic produce. Similarly, Yadav and Pathak [80] found that environmental concerns did not show any significant influence on organic food purchase intention. Overall, AV had the greatest effect on PSe, followed by PV, PS, and RM.

With regards to H3a–c, only H3b gained support (perceived severity—BFB purchase intention, *β* = 0.231, *p* = 0.000), while the other two did not (perceived vulnerability—BFB purchase intention, *β* = 0.073, *p* = 0.081; perceived self-efficacy—BFB purchase intention, *β* = 0.019, *p* = 0.355). Hence, out of the three PMT threat and coping appraisals, only perceived severity significantly predicted the purchase intention towards branded functional beverages. Our observation was in conformity with previous PMT studies [62], whereby perceived severity was confirmed as the strongest predictor of behavioural intention. Simultaneously, our results contradicted those of Park et al. and Cox et al. [59,63], as their findings indicated that self-efficacy was the most important predictor of FF consumption intention. Therefore, future research will be needed that will examine in greater depth the effect of the PMT threat and coping appraisals upon BFB purchase intention.

H4a–c were fully supported by the results obtained (perceived vulnerability—role of media, β = 0.215, *p* = 0.081; perceived severity—role of media, β = 0.111, *p* = 0.023; perceived self-efficacy—role of media, β = 0.135, *p* = 0.006). RM was most influenced by the perceived vulnerability of the Gen Z respondents, followed by PSe and PS. This shows that the perception of COVID-19 related information was most strongly influenced by the sense of vulnerability to COVID-19.

H5 was also fully supported as the three PMT threat and coping appraisal components had a significance positive impact on CB (perceived vulnerability—post-COVID-19 BFB consumption behaviour, β = 0.108, *p* = 0.021; perceived severity—post-COVID-19 BFB consumption behaviour, β = 0.159, *p* = 0.000; perceived self-efficacy—post-COVID-19 BFB consumption behaviour, β = 0.160, *p* = 0.001). PV had the least effect on CB, whereas the effects of PS and PSe on CB were a little stronger. In other words, Gen Z having higher PS and PSe levels demonstrated protective actions (in this case, actual consumption behaviours regarding BFB), which was in conformity with prior PMT studies [81].

Similarly to previous studies [21,44], the BFB purchase intention was found to be a significant antecedent of consumption behaviour in post-COVID-19 times (BFB purchase intention—post-COVID-19 BFB consumption behaviour, *β* = 0.313, *p* = 0.000). Thus, H6 was supported.

H7a–b was fully supported since the results showed that the role of media (information about COVID-19) had a positive effect on the BFB purchase intention (role of media—BFB purchase intention, β = 0.183, *p* = 0.000) and post-COVID-19 BFB consumption behaviour (role of media—post-COVID-19 BFB consumption behaviour, β = 0.140, *p* = 0.002). The impact of RM on PI slightly exceeded the impact of RM on CB. Our results supported the previous findings [24], which revealed that both commercial and human communication hugely affected the way young adults thought and behaved. However, our findings contradicted those of Natarajan et al. [44], as their study did not report any significant influence of the role of media on the purchase intention and consumption behaviour towards branded functional beverages.

Finally, H8a–b were supported, as BFB health benefits positively influenced the BFB purchase intention (BFB health benefits—BFB purchase intention, *β* = 0.221, *p* = 0.000) and COVID-19-related information (BFB health benefits—role of media, *β* = 0.135, *p* = 0.002). BFB health benefits had a stronger impact on the BFB purchase intention than information about COVID-19. Surprisingly, BFB health benefits had an insignificant effect on post-COVID-19 BFB consumption behaviour (BFB health benefits—post-COVID-19 BFB consumption behaviour, β = 0.064, *p* = 0.055), so H8c was rejected. Therefore, Gen Z’s awareness of the health benefits of BFB did not transform into actual activities (i.e., BFB consumption behaviour). Savelli and Murmura [24] (p. 2) contended that “overall, Z-ers are little concerned about healthy eating, and their health education remains secondary to them”. Our observation may also be due to a similar phenomenon.

### 5.2. Mediating Effects

The hypothesised indirect relationships between Z-ers’ egoistic values and the role of media via PMT threat and coping appraisal variables were significant and showed a partial mediation (consumers’ egoistic value orientations—perceived vulnerability—role of media, β = 0.036, *p* = 0.005; consumers’ egoistic value orientations—perceived severity—role of media, β = 0.027, *p* = 0.036; consumers’ egoistic value orientations—perceived self-efficacy—role of media, β = 0.030, *p* = 0.015). Also, PMT threat and coping appraisal variables partially mediated the relation between Z-ers’ altruistic values and the role of media (consumers’ altruistic value orientations—perceived vulnerability—role of media, β = 0.067, *p* = 0.000; consumers’ altruistic value orientations—perceived severity—role of media, β = 0.033, *p* = 0.032; consumers’ altruistic value orientations—perceived self-efficacy—role of media, β = 0.052, *p* = 0.009). Thus, H9 and H10 were supported.

The mediation effect of the PMT threat and coping appraisal variables in the relationship between EV and PI was expected (H11a–c), but the hypothesis was not supported except for H11b. H11b gained support by the data gathered that PS partially mediated the relationship between EV and PI (consumers’ egoistic value orientations—perceived severity—BFB purchase intention, β = 0.056, *p* = 0.000). Hence, H11b was supported, and H11a, H11c were rejected (consumers’ egoistic value orientations—perceived vulnerability—BFB purchase intention, β = 0.012, *p* = 0.106; consumers’ egoistic value orientations—perceived self-efficacy—BFB purchase intention, β = 0.004, *p* = 0.359). In other words, egoistic values had a direct effect on Z-ers’ purchase intention towards branded functional beverages but not an indirect one through the perceived vulnerability and perceived self-efficacy.

When examining the indirect effect of AV on PI again, only PS was a mediating variable, but here, the mediation was full (consumers’ altruistic value orientations—perceived severity—BFB purchase intention, β = 0.069, *p* = 0.000). Thus, H12b was supported, and H12a and H12c were rejected (consumers’ altruistic value orientations—perceived vulnerability—BFB purchase intention, β = 0.023, *p* = 0.095; consumers’ altruistic value orientations—perceived self-efficacy—BFB purchase intention, β = 0.007, *p* = 0.358).

It was noted that the BFB purchase intention significantly and partially mediated the impact of PV and RM on CB (perceived vulnerability—BFB purchase intention—post-COVID-19 BFB consumption behaviour, β = 0.023, *p* = 0.025; role of media—BFB purchase intention—post-COVID-19 BFB consumption behaviour, β = 0.215, *p* = 0.000). Therefore, H13a and H14 were supported, while H13b and H13c were not supported (perceived severity—BFB purchase intention—post-COVID-19 BFB consumption behaviour, β = 0.043, *p* = 0.120; perceived self-efficacy—BFB purchase intention—post-COVID-19 BFB consumption behaviour, β = −0.014, *p* = 0.327). A conclusion could be drawn that individuals with a high level of perceived vulnerability exhibited actual consumption behaviours towards branded functional beverages directly and indirectly through intention. Furthermore, the role of media had both a direct and an indirect effect (through the BFB purchase intention) upon actual consumption behaviours.

It was also noted that the BFB purchase intention fully mediated the impact of HB on CB (BFB health benefits—post-COVID-19 BFB consumption behaviour, β = 0.267, *p* = 0.000). Therefore, H15 was supported. This makes it evident that BFB health benefits did not have a direct effect on actual BFB consumption behaviour but had an indirect one through Z-ers’ purchase intention.

RM significantly mediated the relationships between PMT threat and coping appraisal components and the BFB purchase intention (perceived vulnerability—role of media—BFB purchase intention, β = 0.043, *p* = 0.008; perceived severity—role of media—BFB purchase intention, β = 0.069, *p* = 0.000; perceived self-efficacy—role of media—BFB purchase intention, β = 0.037, *p* = 0.006). The mediation between PV, PSe, and PI was full, whereas that between PS and PI was partial. Hence, H16 was supported. It was found that Z-ers’ BFB purchase intention was not directly affected by the perceived vulnerability and perceived self-efficacy but was indirectly influenced by the PV and PSe via the role of media.

The analysis demonstrated the significant partial mediation role of the information about COVID-19 in the influence of PMT threat and coping appraisal components and HB on CB (perceived vulnerability—role of media—post-COVID-19 BFB consumption behaviour, β = 0.021, *p* = 0.026; perceived severity—role of media—post-COVID-19 BFB consumption behaviour, β = 0.033, *p* = 0.003; perceived self-efficacy—role of media—post-COVID-19 BFB consumption behaviour, β = 0.018, *p* = 0.020). Nguyen and Phan [82] also reported that electronic word-of-mouth information mediated the relationship between COVID-19 virus anxiety and functional food consuming intention.

Finally, RM partially mediated the effect of HB on CB (BFB health benefits—role of media—post-COVID-19 BFB consumption behaviour, β = 0.026, *p* = 0.005). Thus, H17, H18, and H19 were supported. Therefore, RM as a mediator played a prominent role in predicting Z-ers’ intention and actual consumption behaviour towards BFB.

### 5.3. Theoretical and Managerial Implications

As a theoretical contribution, this research adds to previous functional food studies in three ways. Firstly, this study extends the existing knowledge of consumers’ acceptance of branded functional beverages, especially with regards to the cohort we chose, Generation Z. Currently, there is limited research on the antecedents and mediators of BFB consumption behaviour in a post-pandemic setting. Secondly, in order to investigate consumers’ decision-making processes, a research model testing the integration of basic VBN and PMT constructs was proposed. Furthermore, to the best of our knowledge, this is the first study to have investigated the way in which egoistic values and altruistic values impact the PMT threat and coping appraisal factors in a BFB context. The research model was developed in an attempt to provide a comprehensive view of consumption behaviour towards branded functional beverages that takes into account the direct and indirect effects of the factors on the resultant variable. Thirdly, the results of our study confirmed the importance of the role of media as an influential antecedent and mediator of BFB consumption behaviour. No previous research framework is available that includes the impact of the PMT threat and coping appraisal variables on the role of media.

From a business perspective, this research can bring forth several suggestions for policy-makers and marketers in the functional food industry. Firstly, based on our findings that altruistic values were not a determinant of behavioural intentions, a conclusion can be made that special actions need to be implemented in order to reposition the BFB oriented towards environmentally conscious Gen Z through brand associations of FB related to the physical composition of products (e.g., by specifying the natural ingredients, the lack of artificial sweeteners and additives, the minimum processing of the raw materials, the lack of genetically modified crops, etc.) or to the manufacturing process aimed at ecological sustainability. In this sense, marketers and managers in Bulgaria should formulate suitable branding strategies directed to these target consumers and their ecological concerns. Secondly, it was found that certain threat and coping appraisal variables had a positive effect on the BFB consumption behaviour in post-COVID-19 times. Therefore, if other health crises arise in the future, the communication messages should be designed and targeted so as to emphasise the activation of protective behaviour through functional beverage consumption. Thirdly, another important result of our study was that the perceived health benefits of BFB to Gen Z were not transformed into actual behaviour. Therefore, further efforts are needed to promote the empirically established specific health benefits of the bioactive components (responsible for the functionality of functional beverages) in BFB to this generational cohort. This could be achieved via the use of multiple communication channels—that is, through the role of media. Fourthly, although the validation of the process of health-related claims is still rather complex, if the manufacturers of functional beverages have strong scientific evidence of the health effects resulting from the consumption of their products, they could start a health claim authorisation procedure. Such a health claim included in the BFB labelling could raise the consumers’ trust in the announced benefits for their health and affect their purchase decisions [3,8].

## 6. Conclusions and Limitations

Understanding Z-ers’ BFB consumption behaviour is important for various stakeholders, including functional food manufacturers, government agencies, pharmaceutical firms, non-governmental institutions, academics, health food specialists, etc. Our findings showed that the egoistic values (health concern) significantly determined the Z-ers’ purchase intention towards BFB. The role of media proved an important mediator in half of the suggested indirect relationships predicting Z-ers’ intention and consumption behaviour towards BFB. Among the information sources that have an effect on Gen-Z’s healthy eating, social networking services, like Facebook, Instagram, and WhatsApp, are the most trusted (see Appendix A, Table A1). The least trusted sources are newspapers, television programmes and commercials, government agencies, regional directorates, associations of medical specialists in Bulgaria, the WHO, and the European Centre for Disease Prevention and Control. At the communication level, the result on the low rates of trust in the COVID-19 related health recommendations of governmental and non-governmental institutions is of interest to us. A possible explanation may be sought in the decline in the public trust regarding the information released by departments and institutions (involved in the crisis management in the country) in the mass media during the very first wave of the pandemic. As pointed out by Džakula et al. [26], the initial centralised approach to handling the crisis through lockdown made use of the high public trust levels in Bulgaria, but over time, governance became dominated by political and economic considerations. Then, communication to the public became contradictory and the levels of public trust declined dramatically.

Our study has some limitations. Firstly, the data were collected in Bulgaria, which limits the possibility of generalising the results to other countries. Future research could cover other target groups from other populations at different locations. It would also be interesting to collect data on countries that differ in their political, socioeconomic, and cultural characteristics (for instance, developed countries vs. developing countries, countries with collectivist attitudes vs. countries with individualist attitudes, etc.). We focused on the 16–26-year-old subgroup of Generation Z in Bulgaria. All Gen Z representatives could be covered and generalised conclusions could be drawn about the BFB consumption behaviour of the cohort as a whole. Secondly, the results of this study are based on non-probability sampling methods, which may undermine the generalisation of the results. Thirdly, we only tested part of the VBN theory in our model. Therefore, future studies may also include other VBN constructs in their conceptualisation frame. Furthermore, we adopted a holistic approach to BFB by including five product categories in the scope of our study. Future research could be focused on the development and validation of a new scale of BFB consumption behaviour towards one product category—for instance, functional bottled water, which is gaining popularity in Bulgaria.

## Figures and Tables

**Table 1:** Measurement scales for the variables in the research model.

Constructs and Measurement Items	Sources
Egoistic Value (EV) (EV1) I choose food carefully to ensure good health. (EV2) I didn’t consider myself as a health-conscious consumer ^R^. (EV3) I think often about health-related issues.	Yadav (2016) [52] and Sadiq et al. (2022) [49]
Altruistic Value (AV) (AV1) The balance of nature is very delicate and can be easily upset. (AV2) Human beings are severely abusing the environment. (AV3) Humans must maintain the balance with nature in order to survive. (AV4) Human interferences with nature often produce disastrous consequences.	Yadav (2016) [52] and Sadiq et al. (2022) [49]
Perceived Vulnerability (PV) (PV1) There is high probability for someone to contract COVID-19 pandemic. (PV2) I am at risk of getting COVID-19 pandemic. (PV3) COVID-19 pandemic is highly contagious. (PV4) It is possible that I will contract COVID-19 pandemic.	Alhemimah (2023) [61]
Perceived Severity (PS) (PS1) I think COVID-19 pandemic is serious. (PS2) I believe the threat of COVID-19 pandemic is significant. (PS3) I think that COVID-19 pandemic is of high risk. (PS4) COVID-19 pandemic is harmful.	Alhemimah (2023) [61]
Perceived Self-efficacy (PSe) (PSe1) I am confident in my ability to protect myself from getting infected with COVID-19 pandemic. (PSe2) I am confident in the effectiveness of my protection against COVID-19 pandemic. (PSe3) I believe my immune system is strong enough to protect me against COVID-19.	Ryu et al. (2023) [62]
BFB Health Benefits (HB) (HB1) Healthier–less healthy. (HB2) Beneficial for health–harmful to health. (HB3) Good for the immune system–harmful to the immune system. (HB 4) More nutritious–less nutritious. (HB5) Lengthens people’s lifespan–shortens people’s lifespan.	Labrecque et al. (2006) [70]
BFB Purchase Intention (PI) (PI1) I am willing to buy functional beverages while shopping. (PI2) I am willing for online shopping for the purchase of functional beverage products, particularly in the post-pandemic time. (PI3) I will make an effort to buy functional beverages in the near future. (PI4) I will recommend functional beverages to my friends and relatives. (PI5) I intend to buy functional beverages if they are available for sale.	Natarajan et al. (2022) [21,44]
Role of Media (Information about COVID-19) (RM) (RM1) Television programs and commercials influence me to eat and drink healthy. (RM2) Newspapers influence me to eat and drink healthy. (RM3) Social Networking Services like Facebook, Instagram, WhatsApp influence me to eat and drink healthy. (RM4) Health magazines, weeklies, etc. influence me to eat and drink healthy. (RM5) The health recommendations regarding COVID-19 given by government agencies, regional directorates, associations of medical specialists in Bulgaria, the WHO, the European Centre for Disease Prevention and Control, etc., influence me towards healthy eating and drinking. (RM6) I often read COVID related information in the print and electronic media including social networking services.	Natarajan et al. (2022) [44]
BFB Consumption Behaviour Post-COVID-19 (CB) (CB1) I like to consume branded functional beverages. (CB2) I often consume branded functional beverages. (CB3) I know the nutritional content and benefits of consuming branded functional beverages. (CB4) I would prefer to consume branded functional beverages than preparing those items at home. (CB5) I intend to continue consuming branded functional beverages.	Natarajan et al. (2022) [21,44]

Note: ^R^ indicates reverse scoring of items.

**Table 2:** Socio-demographic characteristics of the sample (n = 435).

Variable	Categories	Percentage
Gender	Male	51.5%
	Female	48.5%
Level of education	Higher	9.4%
	Secondary	87.8%
	Primary	2.8%
Personal income	Under 780 BGN	47.4%
	780–1299 BGN	28.7%
	1300–1799 BGN	14%
	1800–2299 BGN	5.1%
	2300 and more BGN	4.8%
Place of residence	Capital	13%
	City above 100 thousand	48.6%
	Town from 50 thousand to 100 thousand	24.7%
	Town from 25 thousand to 50 thousand	12.5%
	Town up to 25 thousand	1.2%

**Table 3:** Reliability and convergent validity.

Construct	Cronbach’s Alpha	CR	AVE
EV	0.851	0.910	0.772
AV	0.787	0.860	0.606
PV	0.913	0.939	0.794
PS	0.877	0.917	0.736
PSe	0.919	0.949	0.861
PI	0.919	0.939	0.757
RM	0.846	0.886	0.565
HB	0.871	0.908	0.664
CB	0.917	0.938	0.751

**Table 4:** Discriminant validity (Fornell–Larcker criterion and HTMT).

Construct	AV	CB	EV	HB	PI	PS	PSe	PV	RM
AV	**0.778**	0.250	0.463	0.091	0.275	0.452	0.543	0.409	0.4
CB	0.224	**0.866**	0.296	0.273	0.576	0.516	0.403	0.467	0.482
EV	0.374	0.264	**0.878**	0.145	0.353	0.397	0.446	0.31	0.338
HB	0.063	0.244	0.13	**0.815**	0.468	0.266	0.377	0.422	0.468
PI	0.245	0.532	0.318	0.315	**0.870**	0.468	0.266	0.377	0.422
PS	0.388	0.466	0.343	0.142	0.427	**0.858**	0.318	0.689	0.426
PSe	0.464	0.371	0.396	0.133	0.245	0.285	**0.928**	0.345	0.373
PV	0.371	0.429	0.278	0.084	0.350	0.618	0.316	**0.891**	0.451
RM	0.333	0.429	0.290	0.200	0.383	0.375	0.332	0.403	**0.752**

The square roots of the AVE values have been marked in bold and presented diagonally. The HTMT values have been exhibited above the diagonal elements.

**Table 5:** Direct relationships.

Hypotheses	Path Coeffects (*β*)	*t*-Statistics	*p*-Value	Decision
H1a. EV→PV	0.166	3.384	0.000	Supported
H1b. EV→PS	0.243	5.255	0.000	Supported
H1c. EV→PSe	0.223	4.657	0.000	Supported
H1d. EV→PI	0.132	2.846	0.002	Supported
H1e. EV→RM	0.091	2.060	0.002	Supported
H2a. AV→PV	0.310	6.700	0.000	Supported
H2b. AV→PS	0.299	6.148	0.000	Supported
H2c. AV→PSe	0.384	8.429	0.000	Supported
H2d. AV→PI	−0.001	0.488	0.294	Not Supported
H2e. AV→RM	0.102	1.905	0.028	Supported
H3a. PV→PI	0.073	1.401	0.081	Not Supported
H3b. PS→PI	0.231	4.403	0.000	Supported
H3c. PSe→PI	0.019	0.371	0.355	Not Supported
H4a. PV→RM	0.215	4.296	0.000	Supported
H4b. PS→RM	0.111	1.989	0.023	Supported
H4c. PSe→RM	0.135	2.531	0.006	Supported
H5a. PV→CB	0.108	2.031	0.021	Supported
H5b. PS→CB	0.160	3.052	0.001	Supported
H5c. PSe→CB	0.159	4.037	0.000	Supported
H6. PI →CB	0.313	5.730	0.000	Supported
H7a. RM→PI	0.183	3.653	0.000	Supported
H7b. RM→CB	0.140	2.905	0.002	Supported
H8a. HB→PI	0.221	5.091	0.000	Supported
H8b. HB→RM	0.135	2.923	0.002	Supported
H8c. HB→CB	0.064	1.594	0.055	Not Supported

Relationships are significant at *p* < 0.05.

**Table 6:** Mediation analysis.

Hypotheses	Path Coeffects (*β*)	*t*-Statistics	*p*-Value	Decision	Mediation Type
H9a. EV→PV→RM	0.036	2.589	0.005	Supported	Partial Mediation
H9b. EV→PS→RM	0.027	1.804	0.036	Supported	Partial Mediation
H9c. EV→PSe→RM	0.030	2.184	0.015	Supported	Partial Mediation
H10a. AV→PV→RM	0.067	3.410	0.000	Supported	Partial Mediation
H10b. AV→PS→RM	0.033	1.854	0.032	Supported	Partial Mediation
H10c. AV→PSe→RM	0.052	2.384	0.009	Supported	Partial Mediation
H11a. EV→PV→PI	0.012	1.246	0.106	Not Supported	No Mediation
H11b. EV→PS→PI	0.056	3.432	0.000	Supported	Partial Mediation
H11c. EV→PSe→PI	0.004	0.360	0.359	Not Supported	No Mediation
H12a. AV→PV→PI	0.023	1.310	0.095	Not Supported	No Mediation
H12b. AV→PS→PI	0.069	3.394	0.000	Supported	Full Mediation
H12c. AV→PSe→PI	0.007	0.364	0.358	Not Supported	No Mediation
H13a. PV→PI→CB	0.023	1.281	0.025	Supported	Partial Mediation
H13b. PS→PI→CB	0.043	1.174	0.120	Not Supported	No Mediation
H13c. PSe→PI→CB	−0.014	0.449	0.327	Not Supported	No Mediation
H14. RM→PI→CB	0.215	6.130	0.000	Supported	Partial Mediation
H15. HB→PI→CB	0.267	6.876	0.000	Supported	Full Mediation
H16a. PV→RM→PI	0.043	2.416	0.008	Supported	Full Mediation
H16b. PS→RM→PI	0.069	3.447	0.000	Supported	Partial Mediation
H16c. PSe→RM→PI	0.037	2.500	0.006	Supported	Full Mediation
H17a. PV→RM→CB	0.021	1.950	0.026	Supported	Partial Mediation
H17b. PS→RM→CB	0.033	2.799	0.003	Supported	Partial Mediation
H17c. PSe→RM→CB	0.018	2.064	0.020	Supported	Partial Mediation
H18. HB→RM→PI	0.053	3.095	0.001	Supported	Partial Mediation
H19. HB→RM→CB	0.026	2.603	0.005	Supported	Partial Mediation

Relationships are significant at *p* < 0.05.

## Data Availability

The data presented in this study are available from the corresponding author upon reasonable request.

## Abbreviations

AV	Altruistic Value
AVE	Average Variance Extracted
BFB	Branded Functional Beverage
BGN	Bulgarian Lev
CB	Branded Functional Beverage Consumption Behaviour Post-COVID-19
CMB	Common Method Bias
CNDs	Chronic Non-communicable Diseases
COVID-19	Coronavirus Disease 2019
CR	Composite Reliability
EV	Egoistic Value
FB	Functional Beverage
FF	Functional Foods
FFC	Functional Centre
FuFoSE	Functional Food Science in Europe
GoF	Goodness of Fit
HB	Branded Functional Beverage Health Benefits
HTMT	Heterotrait–Monotrait Ratio
MUFA	Monounsaturated Fatty Acids
NAM	Norm Activation Model
NSI	National Statistical Institute in the Republic of Bulgaria
PI	Branded Functional Beverage Purchase Intention
PLS-SEM	Partial Least Squares Structural Equation Modelling
PMT	Protection Motivation Theory
PS	Perceived Severity
Pse	Perceived Self-efficacy
PUFA	Polyunsaturated Fatty Acids
PV	Perceived Vulnerability
PVs	Personal Values
RM	Role of Media (Information about COVID-19)
SDG3	United Nations Sustainable Development Goal 3, regarding “Good Health and Well-being”
VBN	Value–Belief–Norm
VIF	Variance Inflation Factors

## Appendix A

**Table A1:** Descriptive statistics of the measurement items.

Items	Mean	St. Deviation	Skewness	Kurtosis
EV1	2.520	1.068	0.251	−0.479
EV2 ^R^	2.400	1.140	0.398	−0.638
EV3	2.730	1.077	0.163	−0.541
AV1	2.750	1.068	0.257	−0.581
AV2	2.330	1.115	0.585	−0.543
AV3	1.800	1.077	1.380	1.163
AV4	1.960	1.167	1.233	0.638
PV1	2.730	1.074	0.147	−0.624
PV2	2.880	1.074	0.020	−0.724
PV3	2.860	1.143	0.054	−0.766
PV4	2.960	1.079	0.027	−0.685
PS1	2.920	1.136	0.102	−0.736
PS2	2.960	1.119	0.043	−0.691
PS3	2.820	1.127	0.071	−0.728
PS4	2.550	1.185	0.461	−0.682
PSe1	2.570	1.035	0.263	−0.461
PSe2	2.600	1.050	0.238	−0.489
PSe3	2.640	1.054	0.116	−0.652
HB1	2.990	1.042	0.075	−0.537
HB2	2.900	1.010	0.145	−0.456
HB3	2.890	1.028	0.219	−0.391
HB4	2.900	1.062	0.136	−0.642
HB5	3.140	1.030	0.174	−0.413
PI1	2.800	1.080	0.079	−0.545
PI2	3.140	1.149	0.117	−0.903
PI3	2.850	1.060	0.143	−0.502
PI4	2.990	1.093	0.214	−0.753
PI5	2.890	1.071	−0.029	−0.743
RM1	2.940	1.069	0.197	−0.561
RM2	3.810	1.217	−0.529	−1.058
RM3	2.870	1.139	0.113	−0.738
RM4	3.150	1.126	0.131	−0.809
RM5	3.060	1.088	0.389	−0.638
RM6	3.630	1.241	−0.503	−0.816
CB1	3.080	1.171	−0.005	−0.829
CB2	3.150	1.120	0.133	−0.633
CB3	3.060	1.190	0.091	−0.852
CB4	3.000	1.098	0.036	−0.731
CB5	3.020	1.178	0.049	−0.778

Note: ^R^ indicates reverse scoring of items.
